# Impaired diversity of the lung microbiome predicts progression of idiopathic pulmonary fibrosis

**DOI:** 10.1186/s12931-018-0736-9

**Published:** 2018-02-27

**Authors:** Youhei Takahashi, Atsushi Saito, Hirofumi Chiba, Koji Kuronuma, Kimiyuki Ikeda, Tomofumi Kobayashi, Shigeru Ariki, Motoko Takahashi, Yasushi Sasaki, Hiroki Takahashi

**Affiliations:** 1 Department of Respiratory Medicine and Allergology, Sapporo Medical University School of Medicine, S1W16 Chuoku, Sapporo, 060-8543 Japan; 2 Department of Biochemistry, Sapporo Medical University School of Medicine, S1W17 Chuoku, Sapporo, 060-8556 Japan; 3 Department of Medical Genome Sciences, Research Institute for Frontier Medicine, Sapporo Medical University, S1W17 Chuoku, Sapporo, 060-8556 Japan

**Keywords:** Idiopathic pulmonary fibrosis, Microbiome, Next-generation sequencing techniques, Bleomycin, Mouse model, Diversity, Disease progression, Surfactant protein

## Abstract

**Background:**

Idiopathic pulmonary fibrosis (IPF) is the most frequent and severe form of idiopathic interstitial pneumonias. Although IPF has not been thought to be associated with bacterial communities, recent papers reported the possible role of microbiome composition in IPF. The roles of microbiomes in respiratory functions and as clinical biomarkers for IPF remain unknown. In this study, we aim to identify the relationship between the microbial environment in the lung and clinical findings.

**Methods:**

Thirty-four subjects diagnosed with IPF were included in this analysis. The 16S rDNA was purified from bronchoalveolar lavage fluid obtained at the time of diagnosis and analyzed using next-generation sequencing techniques to characterize the bacterial communities. Furthermore, microbiomes from mice with bleomycin-induced lung fibrosis were analyzed.

**Results:**

The most prevalent lung phyla were Firmicutes, Proteobacteria and Bacteroidetes. Decreased microbial diversity was found in patients with low forced vital capacity (FVC) and early mortality. Additionally, the diversity and relative abundance of Firmicutes, Streptococcaceae, and Veillonellaceae were significantly associated with FVC, 6-min walk distance, and serum surfactant protein D. Bleomycin-induced lung fibrosis resulted in decrease of diversity and alteration of microbiota in PCoA analysis. These results support the observations in human specimens.

**Conclusions:**

This study identified relationships between specific taxa in BALF and clinical findings, which were also supported by experiments in a mouse model. Our data suggest the possibility that loss of microbial diversity is associated with disease activities of IPF.

**Electronic supplementary material:**

The online version of this article (10.1186/s12931-018-0736-9) contains supplementary material, which is available to authorized users.

## Background

Idiopathic pulmonary fibrosis (IPF) is a well-known life-threatening disease of the lung with median survival of only 3 to 5 years [1–3] Patients with IPF exhibit restrictive ventilatory impairment caused by progressive lung fibrosis, and a decline in forced vital capacity (FVC) is strongly associated with mortality [4]. Among the various forms of idiopathic interstitial pneumonias, IPF has attracted the most attention because of its poor prognosis and the limited number of therapeutic agents. In addition, acute exacerbation (AE) is the most critical life-threatening event in IPF, especially in Asians, including Japanese [5–7]. Many studies have identified key factors in the development of IPF, and some drugs against fibrosis have been developed, but the direct cause remains unknown [8, 9].

Until recently, the lung was considered to be a sterile compartment because culture-based techniques are limited in their ability to identify all bacteria that may be present. However, new techniques have been developed that have identified bacterial communities both in healthy humans and those with pulmonary diseases [10–12]. This new characterization of the lung microbiome is likely to provide important pathogenic insights into chronic lung diseases, including cystic fibrosis, chronic obstructive pulmonary disease, and asthma. Recent papers have reported the possible role of microbiome composition in IPF [13, 14]. Increased bacterial burden and presence of specific bacteria have been associated with disease progression. However, the roles of the microbiome in respiratory functions and as a clinical marker in IPF remain unknown.

In the current study, we performed a retrospective comprehensive analysis of lower respiratory tract bacterial microbiomes in Japanese individuals with IPF. To investigate the clear relationship between microbiome and pathological conditions, we also analyzed microbiome from a mouse model with bleomycin-induced lung fibrosis.

## Methods

### Patient selection

The subjects who were retrospectively included in this analysis were IPF patients who had visited Sapporo Medical University Hospital. The study was approved by the hospital’s ethics board (approval number, 292–35). Diagnosis of IPF was performed according to the ATS/ERS/JRS/ALAT statement 2011 [2]. In this study, drug history (discussed in the Results section) is not included in the exclusion criteria. The patient characteristics, results of pulmonary function tests, arterial blood gas analysis, and 6-min walk distance (6MWD) test, as well as general blood test and some biomarkers for IPF in serum at enrollment were investigated. The pulmonary function test (PFT), including determination of the FVC and diffusing capacity of lung carbon monoxide (DLco), was performed by using a CHESTAC-55 V system (CHEST M.I., Inc., Tokyo, Japan). The 6MWD test was performed in accordance with ATS guidelines. The GAP (gender, age, and physiological variables) point score was calculated by determining the subject’s gender, age in years, percent predicted FVC (%FVC), and percent predicted DL_CO_ (%DL_CO_), as described previously [15]. PFT results at 12 ± 1 months from baseline and the change in FVC were evaluated. Deterioration was defined as a ≥ 10% relative decline in FVC from baseline and severe respiratory status in which PFTs were unable to be performed [16]. AE was defined according to the Japanese criteria [16]. These criteria are consistent with the criteria proposed by Collard and coworkers in 2007 [17].

### Sample collection

Bronchoscopy was performed in patients with clinically diagnosed IPF. The bronchoscope was inserted via the mouth and through the vocal cords. After carefully observing the lumen of the bronchus, the bronchoscope was wedged into a targeted lobe using an intubation tube. Bronchoalveolar lavage (BAL) was performed in either the middle lobe or the lingula, irrespective of the imaging abnormality on high-resolution computed tomography (HRCT). BAL was performed with 3 instillations of 50 ml of warmed normal saline for a total of 150 ml with all possible returns collected and pooled. Samples for microbiome analysis were added to a falcon tube and stored at − 80 °C until the time of DNA extraction.

### Murine model

C57BL/6 J mice were purchased from Sankyo Labo Service Corporation Inc. (Tokyo, Japan). We ensured that they were equivalent in genetic quality and had stable phenotypes. Although non-littermate mice might have different microbial flora, we did not use littermates in this study to avoid a bias arising from the residing genetic inheritance. In addition to similar housing conditions provided to all animals, including food, water and co-house, the random selection ensured elimination of the bias as much as possible. The mice were maintained under specific pathogen-free conditions and used when they were 8 weeks of age. Thereafter, we divided the mice into two groups: bleomycin and normal saline groups. Bleomycin hydrochloride (Nippon kayaku Co., Tokyo, Japan) was dissolved in normal saline and continuously administrated via micro-osmotic pump (Alzet 1007D; DURECT Corporation, CA). Mice were anesthetized by inhalation of isoflurane, followed by Alzet pump insertion into the mid-back subcutaneous region. Alzet pumps contained either 100 μl of bleomycin hydrochloride (100 mg/kg) or normal saline as a control, and were designed to deliver their contents at 0.5 μL/h over 7 days. At 28 days after Alzet pump insertion, mice were sacrificed with an intraperitoneal injection of pentothal, and blood was immediately collected for obtaining serum samples. Lungs were lavaged with 1 ml of normal saline. For histological examination, lungs were fixed with 10% formalin phosphate and embedded in paraffin. Lung sections were stained with hematoxylin-eosin and Masson trichrome reagent, and examined with a microscope. Total and differential cells in the bronchoalveolar lavage fluid (BALF) were counted using the XT-1800iV (Sysmex, Japan). All experiments using mice were conducted according to the regulations of the Sapporo Medical University Animal Care and Use Committee.

### Standard lysis protocol and DNA isolation

A 1.5 ml aliquot of the BALF sample was centrifuged at 10,000 *g* for 10 min, and the supernatant was discarded. The pellets were suspended in 180 μl of lysis solution (20 mg/ml egg chicken lysozyme (Sigma-Aldrich), 20 mM Tris-HCl (pH 7.4), 2 mM EDTA, 1.2% Triton X), then incubated for 30 min at 37 °C with occasional vortex mixing. Samples were digested by Proteinase K (final concentration, 1 mg/mL) and incubated for 30 min at 65 °C. DNA purification was performed using a DNeasy Blood and Tissue Kit (Qiagen) per manufacturer instructions.

### Real-time polymerase chain reaction (PCR)

The number of tuf gene expression was analyzed to estimate the number of bacteria in the BALF. Real-time PCR was performed on these samples by using a bacterial quantitative PCR kit (Takara, Japan) and a 7900 HT Fast Real-Time PCR System (Applied Biosystems). The cycling conditions were 1 cycle at 95 °C for 30 s, 35 cycles at 95 °C for 5 s, and 60 °C for 30 s.

### DNA sequencing and sequence analyses

An Ion 16S Metagenomics Kit, which is designed for rapid analyses of bacterial samples using Ion Torrent sequencing technology, was used for the sequencing analysis of the bacterial 16S rDNA gene. This kit includes 2 primer sets that selectively amplify the corresponding hypervariable regions of the 16S region in bacteria: V2–4-8 and V3–6, 7–9. The primary PCR cycling conditions were 95 °C for 10 min, followed by 27 cycles of standard PCR (95 °C for 30 s, 50 °C for 30 s, and 72 °C for 45 s), and finished with 72 °C for 7 min. Then, sequence analyses were performed by using the Ion Torrent sequencing platform (Ion Torrent PGM™). Raw sequencing data were processed by using the mother software package, as described in the Ion Torrent standard operating procedure. After sequencing, raw signal data were analyzed using Torrent Suite version 5.0. The pipeline included signaling processing, base calling, low quality read removal, and adapter trimming. Sequencing data in BAM format were further processed using Ion Reporter software v5.2. These files, with the taxonomic information for the operational taxonomic units (OTUs), were imported and further analyzed using Explicet v2.10 software (Robertson, http://www.explicet.org). We restricted the principal components and regression analyses to OTUs that were present at > 1.0% of a given sample’s population and taxonomically identifiable.

### Statistical analysis

Continuous variables are shown as the median with interquartile ranges. For comparisons between two groups, continuous variables were examined by using the Mann–Whitney U test on GraphPad Prism v7 software (GraphPad, Inc., San Diego, CA, USA). Alpha-diversity metrics were used to characterize the bacterial diversity within each sample. Alpha-diversity metrics, including the Shannon diversity index and Simpson diversity (1-D) index, were computed by using Explicet v2.10 software. Spearman’s correlation analysis and JMP 13.0 (SAS Institute, Cary, NC, USA) were used to evaluate the coefficients of determination (ρ), residuals, and significance (p) to identify associations between the microbiome and parameters. Principal coordinates analysis (PCoA) of the Bray-Curtis dissimilarity between samples was generated using Past v3.13 software (Hammer O. Past 3.× 2017. Available from: http://folk.uio.no/ohammer/past/).

## Results

### Patient characteristics and clinical data

The subjects included in this analysis are shown in Table 1. Thirty-four patients with suspected IPF were enrolled in this study from July 2013 to December 2015 and underwent bronchoscopy. A diagnosis of IPF was made by ≥3 pulmonologists on the basis of HRCT. All 34 patients had a consistent usual interstitial pneumonia pattern. None of 34 patients received surgical lung biopsy for definitive diagnosis. The 34 subjects with IPF were predominantly men (73.5%). The median age was 69 years old. According to the GAP score, 22 patients had stage I, 8 had stage II, and 4 had stage III disease, and the subjects were considered to have had mild or moderate disease at enrollment. The incidence of an AE within 12 months from entry was 14.7%. There were 4 deaths reported among the 34 study participants in the initial 12 months. Before the time of assessment, the participants were undergoing treatment with clarithromycin (*n* = 1), inhaled corticosteroids (n = 1) or antacids (*n* = 10), respectively. None of the participants were taking systemic immunosuppressants, N-acetylcysteine or antifibrotic agents at the time of entering this study. During the observation period, the participants received treatment with antifibrotic agents (*n* = 7), systemic corticosteroids (*n* = 4), cyclophosphamide (*n* = 2) and N-acetylcysteine (*n* = 5) at some point. Table 1 also summarises the information of the total cell counts in BALF. Although the alteration in alveolar microbial flora is often accompanied by changes in BALF cell counts, no correlation was established between BALF cell counts and the microbiome in this study.

**Table 1:** Baseline characteristics of the study patients

Age (years)		69 (63–72)
Sex	M	25 (73.5%)
	F	9 (26.5%)
Smoking History	current or ex	27 (79.4%)
	never	7 (20.6%)
GAP score	I	22 (64.7%)
(Stage)	II	8 (23.5%)
	III	4 (11.8%)
SP-A (ng/ml)		77.0 (43.3–95.2)
SP-D (ng/ml)		217.0 (150.0–333.0)
KL-6 (U/ml)		972.0 (572.0–1440.0)
PaO_2_ (mmHg)		87.8 (80.7–91.6)
PaCO_2_ (mmHg)		40.2 (37.6–41.7)
AaDO_2_		12.6 (8.0–17.9)
FVC (L)		2.7 (2.1–3.2)
%FVC		93.0 (75.5–104.3)
FEV1 (L)		2.3 (1.9–2.6)
%FEV1		83.3 (76.9–88.2)
DLCO (ml/min/mmHg)		12.0 (10.1–15.4)
%DLCO		60.0 (52.4–76.1)
6MWD (m)		430 (365–480)
Minimum SpO_2_ (%) during 6MWT		93 (88–99)
Cell Recovery (×10^5^/ml)		1.23 (0.65–1.67)
Cell population (%)		
Macrophages		87.4 (81.8–92.9)
Lymphocyte		6.2 (2.7–11.8)
Neutrophils		2.2 (1.4–3.6)
Eosinophils		1.55 (0.4–3.1)
CD4/8 (ratio)		2.0 (1.1–2.7)

n (%) or median ± IQR. DLCO, %DLCO; *n* = 31, other; *n* = 34. Definition of abbreviations: *FVC* Forced vital capacity, *%FVC* Percent predicted FVC, *FEV1* Forced expiratory volume in the first second, *%FEV1* Percentage of predicted FEV1, *DLco* Diffusing capacity of lung carbon monoxide, *%DLco* Percent predicted DL_CO_, *SP-A* Surfactant protein A; *SP-D* Surfactant protein D; *KL-6* Krebs von den Lungen-6; *6MWD* 6-min walk distance; *6MWT* 6-min walk test

### The diversity of the lung microbiome was associated with progression of IPF.

**Fig. 1:**
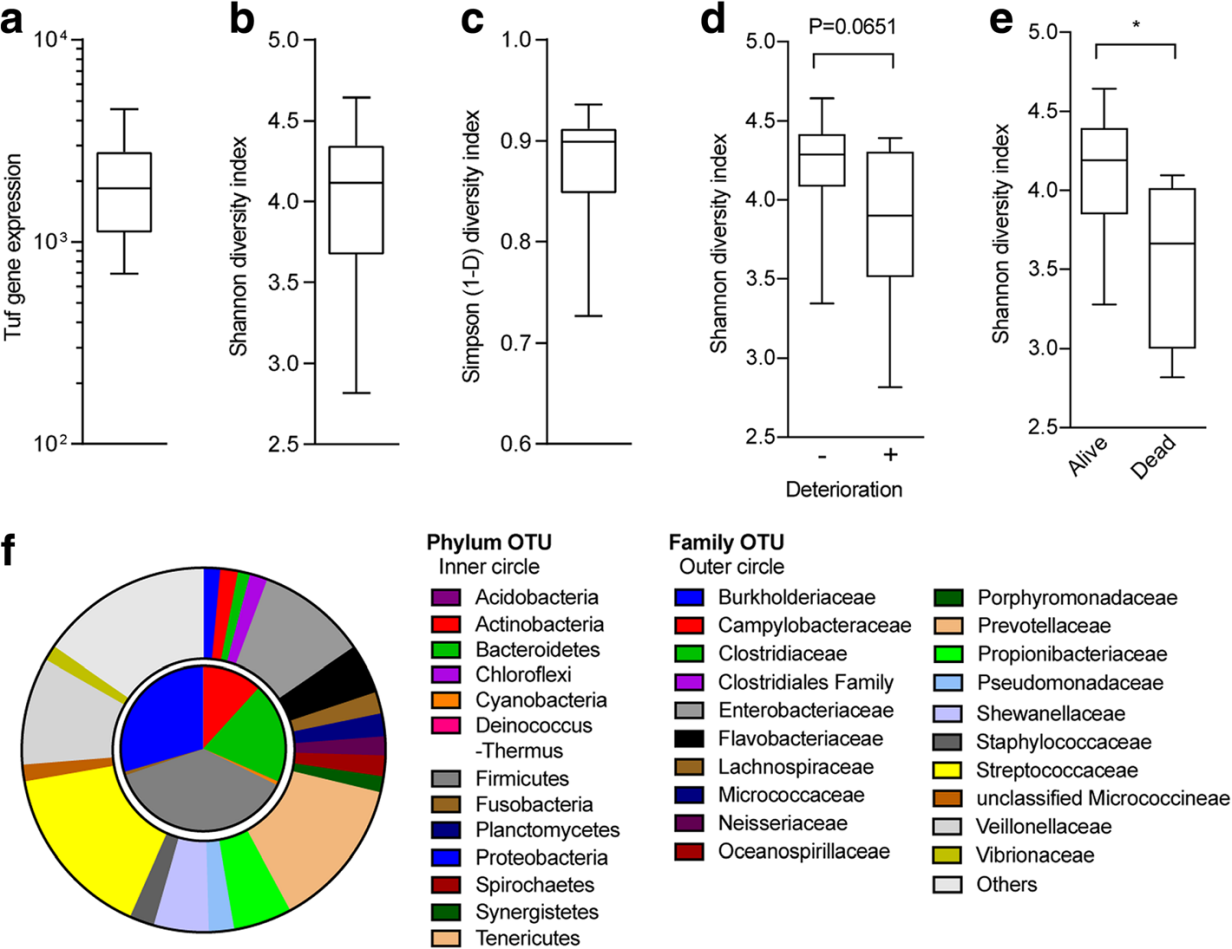
**a** Total number of tuf gene was measured by real-time PCR. **b**, **c** The alpha diversity is shown by a box plot. **d** Shannon diversity index is compared between IPF with or without deterioration. **e** IPF patients who died within 12 months had a significantly lower Shannon diversity index. Box plots are expressed as the median ± IQRs, Mann–Whitney U test, *: *p* < 0.05. **f** Phylum and family level of operational taxonomic units from 16S rRNA gene sequences. Microbial subjects with > 1.0% relative abundance per sample are shown

The number of tuf gene was measured instead of the number of bacteria. Figure 1a shows the overall total bacterial load measured in 1 ml of BALF, and values ranged from 601 to 4554 (Fig. 1a). There was no statistically significant difference regardless of the history of AE (Additional file 1: Figure S1). Overview of DNA sequencing data are shown in Additional file 1: Table S1. The number of OTUs and sequences reads per BALF sample ranged from 56 to 93 and from 29,956 to 132,216 in the family level classification, respectively. Rarefaction curves were calculated to measure how well the amount of sequencing performed for a library represented the biodiversity in the library, and the measured values ranged from 0%–100%, with 100% indicating that all expected OTUs were observed, which would suggest that deeper sequencing would be unlikely to identify additional OTUs. The rarefaction curves in this analysis indicated that sufficiently deep sequencing had been achieved (Additional file 1: Figure S2). To evaluate the microbial diversity in these subjects, the Shannon and Simpson diversity indexes were examined. The values in the Shannon diversity index ranged from 2.817 to 4.623. Poor OTU diversity was obtained in three patients, which had values of 2.817, 3.281, and 3.347. The Simpson diversity index ranged from 0.727 to 0.931, which were parallel to the indices of the Shannon diversity index (Fig. 1b and c). To examine whether the diversity of the pulmonary microbiome influenced disease progression, we compared the diversity with the clinical data of 30 patients in the non-deterioration group (*n* = 22) and the deterioration group (*n* = 8; 7 had a − 10% or greater decrease in FVC and 1 could not perform the PFT) (Fig. 1d). In all patients within the deterioration group (n = 8), the diversity index tended to be smaller than that in the patients of the non-deterioration group. Additionally, the diversity index was significantly smaller in the early death populations than in the survival populations (Fig. 1e). These results suggested the possibility that the diversity of the pulmonary microbiome could be used to predict prognosis for IPF pathogenesis. The relative abundances of bacterial phyla and family OTUs with > 1.0% relative abundance per sample are shown in Fig. 1f. The most dominant lung phyla found were Firmicutes, Proteobacteria, Bacteroidetes, and Actinobacteria (37.4%, 29.4%, 19.8%, and 11.7%, respectively). Additionally, we observed Cyanobacteria and Fusobacteria in the lung. The largest and second largest family OTU in the Firmicutes were the Streptococcaceae and Veillonellaceae (14.9% and 9.7%, respectively). The second largest family OTU was Prevotellaceae (12.1%), which is a member of the Bacteroidetes. Other common OTUs identified in the family included Propionibacteriaceae, Shewanellaceae, Pseudomonadaceae, and Staphylococcaceae. Considering the diversity more deeply, we presumed that the decrease in diversity was due to an increase in Firmicutes and a decrease in Proteobacteria. Particularly concerning Firmicutes, involvement of the Streptococcaceae and Veillonellaceae families was shown (Table 2).

**Table 2:** The relationships between diversity and the relative abundance of bacteria

	Shannon diversity index		Simpson diversity index	
	ρ	*p*-value	ρ	*p*-value
vs Firmicutes	−0.5696	0.0004 **	−0.4599	0.0062 **
vs Proteobacteria	0.7635	< 0.0001 **	0.6468	< 0.0001 **
vs Bacteroidetes	−0.3744	0.0292 *	−0.2567	0.1427
vs Streptococcuceae	−0.3368	0.0515	−0.3035	0.0810
vs Veilonellaceae	−0.3517	0.0413 *	−0.2180	0.2154
vs Prevotellaceae	−0.4359	0.0100 *	−0.2656	0.1290

These OTUs had a strong impact on diversity in the lung. Spearman’s test. ρ: Spearman rank-correlation coefficient, *: p < 0.05, **: p < 0.01

### Micro-organisms were associated with the clinical biomarkers or physiological assessment.

**Fig. 2:**
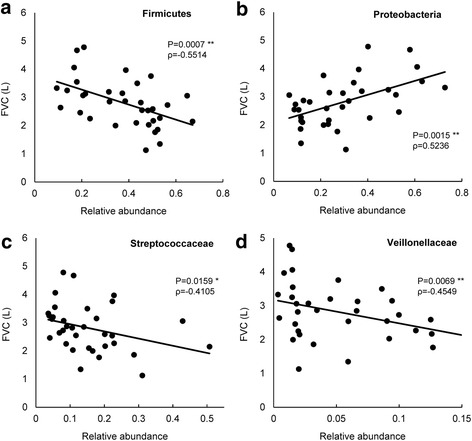
The relationships between FVC and relative abundance of bacteria. **a** Phylum Firmicutes, (**b**) Phylum Proteobacteria, (**c**) Family Streptococcaceae and (**d**) Family Veillonellaceae. In each case, linear regression lines have been fitted. Spearman’s test. ρ: Spearman rank-correlation coefficient, *: p < 0.05, **: *p* < 0.01

**Fig. 3:**
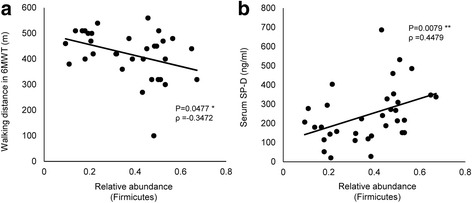
Scatter plots show the correlations of the relative abundance of Firmicutes with (**a**) 6MWD and (**b**) serum SP-D. In each case, linear regression lines have been fitted. Spearman’s test. ρ: Spearman rank-correlation coefficient, *: p < 0.05, **: p < 0.01

Next, we evaluated correlations between clinical data and microbiomes. The FVC was used as an index to understand the progress of IPF, and we first focused on the relationship between the FVC and pulmonary microbiome. We observed no significant association between bacteria burden and FVC (ρ = − 0.153; *P* = 0.395). However, we found that the FVC decreased in inverse proportion to the relative abundance of Firmicutes. On the other hand, the relative abundance of Proteobacteria closely paralleled the FVC results. At the family level, the relative abundances of Streptococcaceae and Veillonellaceae, the largest and second largest single OTU in a member of the Firmicutes, was significantly associated with a decline in FVC (Fig. 2a–d). Furthermore, the Shannon diversity index was positively correlated with the FVC (Table 3). The diversity index from microbial communities are also correlated to the serum level of surfactant protein D (SP-D), lactate dehydrogenase (LDH), and 6MWD, which are the clinical biomarkers or physiological assessment of progression in IPF (Table 3). The families of the Firmicutes phylum also correlated with 6MWD and serum level of SP-D (Fig. 3a and b). Bacterial burden demonstrated an inverse correlation with 6MWD (ρ = − 0.347; *P* = 0.051). However, we established no other obvious relationship between bacterial burden and the clinical biomarkers or physiological assessment. Taken together, these results showed that a reduction in diversity caused by an increase in Firmicutes (Streptococcaceae and Veillonellaceae families) and a decrease in Proteobacteria correlated with disease worsening, which indicated a possible contribution of these organisms to IPF pathogenesis.

**Table 3:** Diversity statistics of pulmonary microbiomes with clinical findings

	Shannon diversity index		Simpson diversity index	
	ρ	*p*-value	ρ	*p*-value
vs FVC (L)	0.4280	0.0116 *	0.3757	0.0286 *
vs %FVC (%)	0.4948	0.0029 **	0.4154	0.0145 *
vs 6MWD (m)	0.3716	0.0332 *	0.3509	0.0452 *
vs SP-D (ng/ml)	− 0.4230	0.0127 *	− 0.4538	0.0070 **
vs LDH (IU/L)	−0.3283	0.0580	−0.3471	0.0443 *

Spearman’s test. ρ: Spearman rank-correlation coefficient, *: p < 0.05, **: p < 0.01

### Microbiome was altered in bleomycin-induced lung fibrosis.

**Fig. 4:**
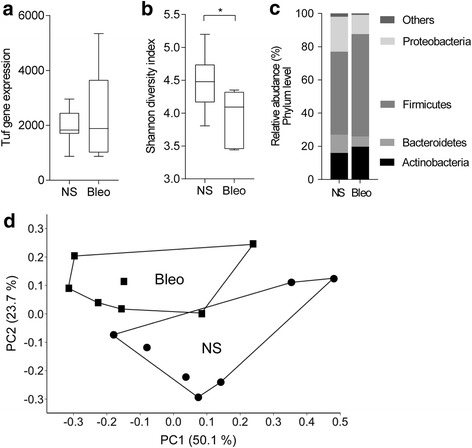
Bleomycin-treated fibrosis alters microbiome in mice lung. C57BL/6 J mice were treated with bleomycin (Bleo) and normal saline (NS) solution as described in methods. **a** Total number of tuf gene in BALF was measured by real-time PCR. **b** The alpha diversity was shown by a box plot. Box plots are expressed as the median ± IQRs. Mann–Whitney U test. *: p < 0.05. **c** Phylum level of operational taxonomic units from 16S rRNA gene sequences. Microbial subjects with > 1.0% relative abundance per sample are shown. **d** Two-dimensional PCoA plot of microbial communities in lung from bleomycin- and saline-treated mice, indicated as significant by PERMANOVA (*p* = 0.0209). Axes show percentage of contribution, PC1: 50.1%, PC2: 23.7%

Microbiomes are thought to be affected by various confounding factors such as smoking history, drugs, etc. [18]. To investigate the clear relationship between microbiome and pathological conditions, we examined the microbiome in mice with the bleomycin-induced lung fibrosis. BAL was performed at 28 days post bleomycin, then representative photomicrographs are presented in Additional file 1: Figure S5. Histopathological assessment showed that diffuse intra-alveolar fibrosis and focally dense fibrosis at the sub-pleural region resulted in increases in the number of cells, especially alveolar macrophages, in BALF (Additional file 1: Figure S5). Although the degree of fibrosis was slightly different in individual mice, all mice treated with bleomycin were included in this analysis, irrespective of the degree of fibrosis. There was no difference in total load of tuf gene between the bleomycin-treated and saline-treated mice (Fig. 4a). Conversely, the Shannon diversity index was significantly smaller in bleomycin-treated mice compared to saline-treated mice. (Fig. 4b). The most dominant lung phyla found were Firmicutes, Proteobacteria, Bacteroidetes, and Actinobacteria in saline-treated mice lungs (49.9%, 20.9%, 10.9%, and 16.0%, respectively). In particular, a significant increase was confirmed in Firmicutes in BALF from bleomycin-treated mice (*p* < 0.05) (Fig. 4c). These results indicate that the alternation of the microbiomes in phylum on fibrotic changes is quite similar between the human samples and mice. Principal coordinate analysis (PCoA) was performed to establish relationships among all samples. The PCoA graph demonstrated that the OTUs detected in BALF of bleomycin-treated mice were well separated from that of saline controls, which indicates that the microbial communities found in bleomycin-treated mice were different from those in the saline controls (Fig. 4d).

## Discussion

In this study, we showed that BALF from initially diagnosed IPF contained microbiota and that the loss of diversity of the lung microbiome correlated with the progression of IPF. Regarding the change in lung microbiome diversity, we found that increases in the Firmicutes and Bacteroidetes (Streptococcaceae, Veillonellaceae, and Prevotellaceae families) and a decrease in the phylum Proteobacteria were involved in the reduction of diversity. Moreover, the loss of diversity correlated with IPF indicators, including low FVC, decreased 6MWD, and high serum SP-D and LDH. Unfortunately, there were no healthy controls in this study, but loss of diversity in IPF compare to healthy control was reported in a previous paper [13]. Also, Molyneaux PL et al. suggested that the bacterial communities of the lower airways act as persistent stimuli for repetitive alveolar injury [19]. Taken together, loss of diversity might have some impact on the pathogenesis of IPF. There is also a possibility that diversity is related to the mortality rate and FVC decline 1 year after diagnosis, which suggests that lung microbiome diversity may be a prognostic indicator, which is the most crucial finding in this study. Furthermore, racial differences and environmental factors were also considered to be involved in the microbiome and obtaining similar results in Asia compared with Western countries was significant. Additionally, we found that an increase in Streptococcaceae correlated with a reduction in 6MWD (Additional file 1: Figure S4). These results possibly suggest that pulmonary microbiomes and lung fibrosis are closely related and are involved in the pathogenesis of IPF. In contrast, whether microbial changes are a cause or merely a consequence of disease progression remains unclear. Hence, further extensive interventional studies, including antibiotics treatment or vaccines to alter the lung microbiome, are needed to elucidate this phenomenon.

Meanwhile, the examinations of microbiomes using clinical samples are quite complicated, because there are many confounding factors such as smoking history, drugs, etc. So, we decided to verify our results of the clinical samples using the bleomycin-treated mice with a uniform background, which is widely used in IPF studies. Since the living environment of the mice is very different from humans, their detailed bacterial flora was expected to be different [20]. However, we found that the relative changes in the bacterial communities accompanying fibrosis in the mice were quite similar to that in IPF patients (Fig. 4c). The increase in the relative abundance of Firmicutes and the decrease in that of Proteobacteria, and its accompanying reduction in diversity were observed in BALF from bleomycin-treated mice (Fig. 4b and c). These results partly supported the analysis in the human specimens. Furthermore, PCoA analysis in the mice revealed that the microbial communities under bleomycin-induced fibrosis were different from normal flora (Fig. 4d), which is not detected in human subjects (data not shown). This may be due to the fact that there are many confounding factors among the patients as mentioned above. It is still difficult to identify causative bacteria implicated in the progression of IPF. Nonetheless, our data suggest that fibrotic changes in the lungs are related to the alternations of specific bacteria or the decline of diversity.

The Hokkaido study revealed that the most common (40%) cause of death was an AE, which resulted in rapid disease progression and a mortality rate of 80% [7]. As the causes of AE, including whether or not bacteria are involved, have not been determined, we believe that it is essential to prove the effect of microbiomes on AEs. Based on our examination at diagnosis, the occurrence of an AE in the future was not related to an increased total bacterial burden and the loss of diversity (Additional File 1: Figure S1). However, because this study highlighted the possibility of the effect of microbiomes on AEs [21–23], we intend to elucidate these findings in our next larger prospective study. Furthermore, we believe that it is imperative to investigate the causative bacteria on fibrosis in a murine model in the future.

An imbalance in the host–microbial relationship is thought to contribute to inflammation and immunity in the IPF lung [24]. It is well known that the pulmonary collectins, surfactant proteins A and D, have an important role in the alveolar spaces and are useful biomarkers in IPF [25]. In this study, serum SP-D was strongly associated with microbiome characteristics, including diversity, the relative abundance of Firmicutes, Proteobacteria, and Veillonellaceae. However, serum SP-A correlated poorly with lung microbiomes in contrast to BALF SP-A (Additional file 1: Figure S3). Our previous study reported that serum levels of SP-D could reflect pathological changes of IPF lungs more decisively than those of SP-A [26]. Because the correlation between BALF SP-A and Veillonellaceae is positive, SP-A production from alveolar type II cells is thought to be increased by inflammatory stimulation accompanying the increase in bacteria. Taken together, it is possible that serum SP-A, in contrast to BALF SP-A, did not correlate with the microbiomes because SP-A remains bound to surfactant lipids in the alveolar space. On the other hand, SP-D reflects changes in the microbiome, such as diversity, because SP-D easily leaks into the bloodstream. As described above, because pulmonary collectins have an important role in pulmonary environments, we plan to clarify the association between the lung microbiome and the collectins in a future study.

This study had some limitations. All participants in this study satisfied the diagnostic criteria of IPF; however, because surgical lung biopsy was not performed at the time of diagnosis, the possibility of including other similar fibrotic diseases cannot be ruled out. In addition, the number of participants (*n* = 34) was small to evaluate the association between the microbiome and IPF pathogenesis, because there are many confounding factors among IPF participants. For this reason, the examination using mice was carried out to prove the results of the human specimens by avoiding the influence of the confounders, although the etiology is somewhat different from IPF. Mice study cannot prove or disprove human studies because of their distinctly different nature and environment; it can provide credence to human studies, but cannot prove it. Furthermore, the inclusion of healthy controls would have been ideal, but there were no healthy controls in this study. Future studies using a larger number of subjects with appropriate controls are needed to further investigate the role of the microbiome in IPF. Additionally, the presence or absence of emphysematous change and the degree or location of fibrosis was not considered in this study. It is possible that the microbiomes may change depending on the grade of fibrosis and honeycomb change. Previous studies have shown that patients with some respiratory past history have different microbiomes than those of healthy volunteers [27]. Furthermore, antibiotics, antacids, inhaled corticosteroids and nutritious food, which can cause microbial changes, should be considered. Because the possible effect of antacids or inhaled corticosteroids on the microbiome cannot be denied in this study. Technically, BAL was carefully performed to prevent contamination from the upper respiratory tract because sampling of the airways by bronchoscopy depends on inserting the bronchoscope into the mouth. It is still possible that contamination from the upper respiratory tract was present; however, a previous report indicated that contamination with oral microbiota from use of typical bronchoscopy techniques was limited [28].

## Conclusions

In summary, this study identified relationships between the relative abundance of specific taxa in BALF and clinical findings, such as the FVC, 6MWD, and serum SP-D. Previous reports have shown that microbes were associated with IPF progression. Our data indicated that the diversity of the pulmonary microbiomes might be a prognostic factor of IPF pathogenesis. Bleomycin-induced lung fibrosis in mice revealed the association between microbiome and fibrotic changes, which also supported the results in the human samples. Further study of the lung microbiome may provide novel insights into its possible contribution to IPF pathogenesis and help identify new biomarkers.

## Additional file

Additional file 1: Impaired Diversity of the Lung Microbione Predicts Progression of Idiopathic pulmonary Fibrosis. **Table S1**. Overview of DNA sequencing data. **Figure S1**. Comparison of the total bacterial genes between the AE and non-AE groups. **Figure S2.** Rarefaction curves were calculated, which suggests that all expected OTUs have been obsreved. **Figure S3**. the concentration of SP-A in BALF correlates well with the relative abundance of Veillonellaceae, but not serum SP-A. **Figure S4**. The relative abundance of family Streptococcaceae also correlated with 6MWD. **Figure S5**. Histopathological assesment and BAL cell counts in mice with the bleomycin-induced lung fibrosis. (PDF 7772 kb)

## Abbreviations

%DLco: Percent predicted DL_CO_
%FEV1: Percentage of predicted FEV1
%FVC: Percent predicted FVC
6MWD: 6-min walk distance
AE: Acute exacerbation
BAL: Bronchoalveolar lavage
BALF: Bronchoalveolar lavage fluid
DLco: Diffusing capacity of lung carbon monoxide
FEV1: Forced expiratory volume in the first second
FVC: Forced vital capacity
HRCT: High-resolution computed tomography
IPF: Idiopathic pulmonary fibrosis
OTU: Operational taxonomic units
PFT: Pulmonary function test
SP-A and D: Surfactant protein A and D
