# Supplementary material for: Impaired diversity of the lung microbiome predicts progression of idiopathic pulmonary fibrosis
Source: Respir Res. 2018 Feb 27;19:34. doi: 10.1186/s12931-018-0736-9 (PMC6389110; doi:10.1186/s12931-018-0736-9)

ADDITIONAL FILE

Impaired Diversity of the Lung Microbiome Predicts Progression of Idiopathic Pulmonary Fibrosis

Youhei Takahashi, MD, Atsushi Saito, MD, PhD, Hirofumi Chiba, MD, PhD, Koji Kuronuma, MD, PhD, Kimiyuki Ikeda, MD, Tomofumi Kobayashi, MD, Shigeru Ariki, PhD, Motoko Takahashi, MD, PhD, Yasushi Sasaki, MD, PhD and Hiroki Takahashi, MD, PhD

RESULTS

Table S1

Figure S1

Figure S2

Figure S3

Figure S4

Figure S5

RESULTS

Table S1. Overview of DNA sequencing data.

No.	Number of sequences	OTU's	Shannon	Simpson 1-D
1	110507	56	3.551	0.850
2	77345	56	3.347	0.786
3	55954	76	4.411	0.910
4	112968	73	3.594	0.843
5	77465	60	3.281	0.727
6	82966	83	4.251	0.879
7	95462	77	4.331	0.922
8	124723	82	4.411	0.917
9	74019	66	3.906	0.867
10	128326	81	4.289	0.910
11	105223	83	4.233	0.906
12	109226	90	4.063	0.867
13	67001	60	3.497	0.850
14	55782	54	3.665	0.844
15	104216	61	4.086	0.910
16	40089	70	4.437	0.917
17	136355	74	4.110	0.906
18	73021	85	4.393	0.911
19	108241	79	4.126	0.903
20	115102	77	4.096	0.894
21	67413	69	3.676	0.820
22	102943	75	4.027	0.886
23	134002	68	2.817	0.729
24	99271	80	4.327	0.900
25	132216	85	4.323	0.916
26	75136	85	3.777	0.844
27	65428	85	4.643	0.936
28	75670	93	4.151	0.880
29	84220	90	4.515	0.929
30	124375	82	4.083	0.899
31	86017	73	4.424	0.925
32	29956	47	3.649	0.840
33	136339	83	4.293	0.911
34	77948	80	4.632	0.931

Figure S1. Comparison of the total bacterial genes between the AE and non-AE groups. There is no statistical difference, but the median of the AE group is slightly higher than that of the non-AE group. Box plots are expressed as the median ± IQRs, p=0.25, Mann–Whitney U test.

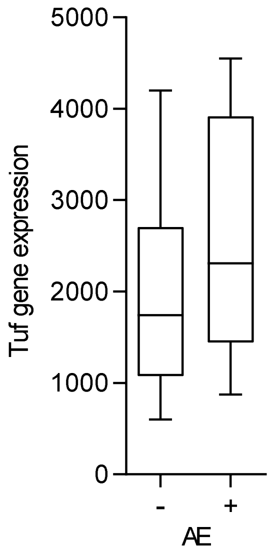

Figure S2. Rarefaction curves were calculated, which suggests that all expected OTUs have been observed.

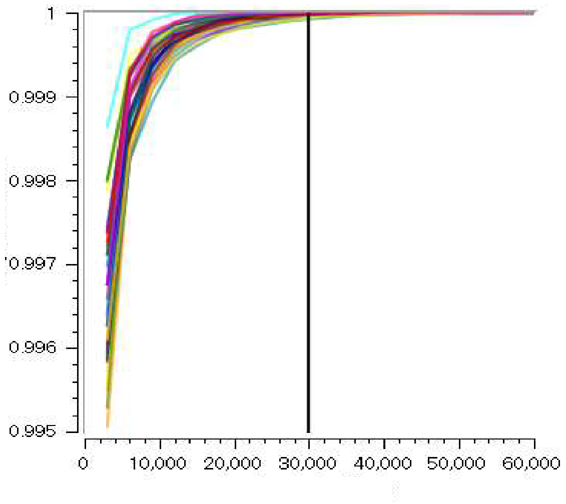

Figure S3. The concentration of SP-A in BALF correlates well with the relative abundance of Veillonellaceae, but not serum SP-A. P=0.0184 and 0.9051, ρ=0.4022 and -0.0212, respectively. Spearman's test. ρ: Spearman rank-correlation coefficient.

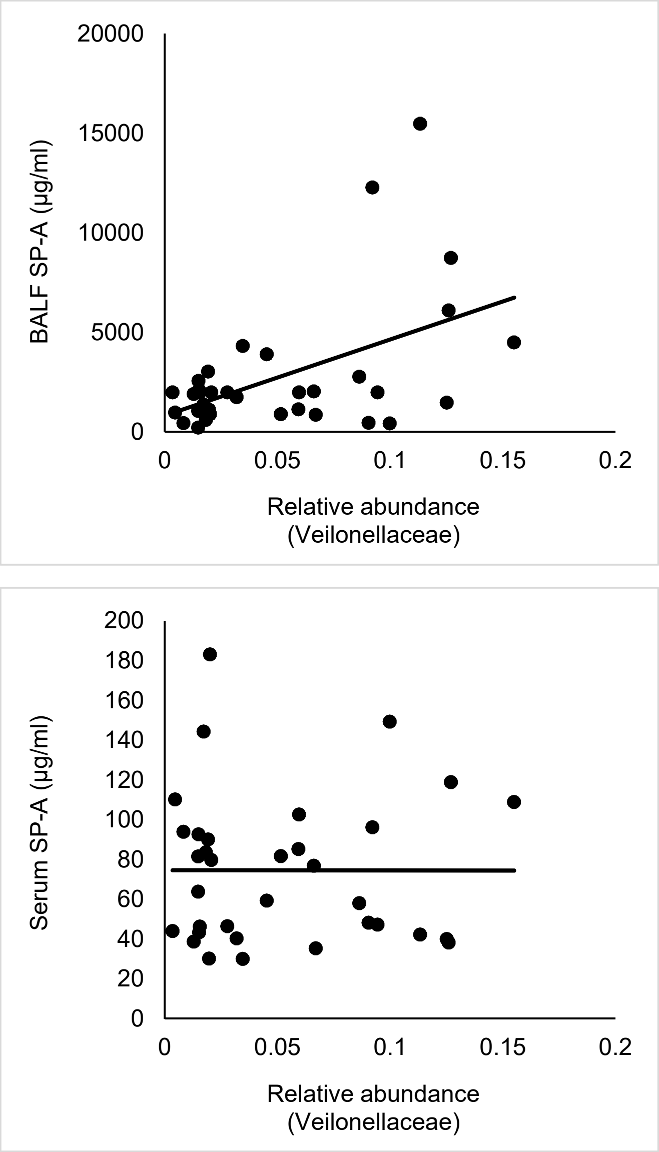

Figure S4. The relative abundance of family Streptococcaceae also correlated with 6MWD. P=0.0030, ρ=-0.5008. Spearman's test. ρ: Spearman rank-correlation coefficient.

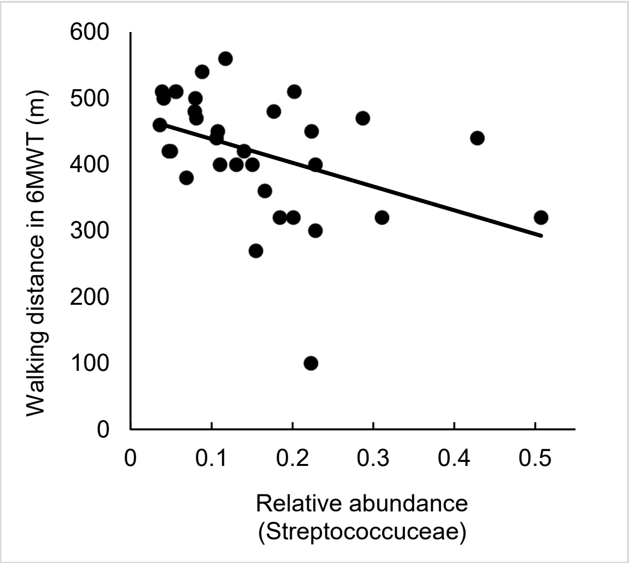

Figure S5. Histopathological assessment and BAL cell counts in mice with the bleomycin-induced lung fibrosis. (A) Lung sections from bleomycin-treated mice were stained with Hematoxylin-Eosin and Masson's trichrome reagent. Representative images are shown. (B, C) The number of total cells and differential cells in bronchoalveolar lavage fluid from bleomycin- (Bleo) and saline-treated (NS) mice were counted. Neutrophils (Neut), Lymphocytes (Lymph), Macrophages (Mac), Eosinophils (Eo) and Basophiles (Baso). Box plots are expressed as the median ± IQRs, (n=6 mice per group), Mann–Whitney U test, *: p<0.05, **: p<0.01.

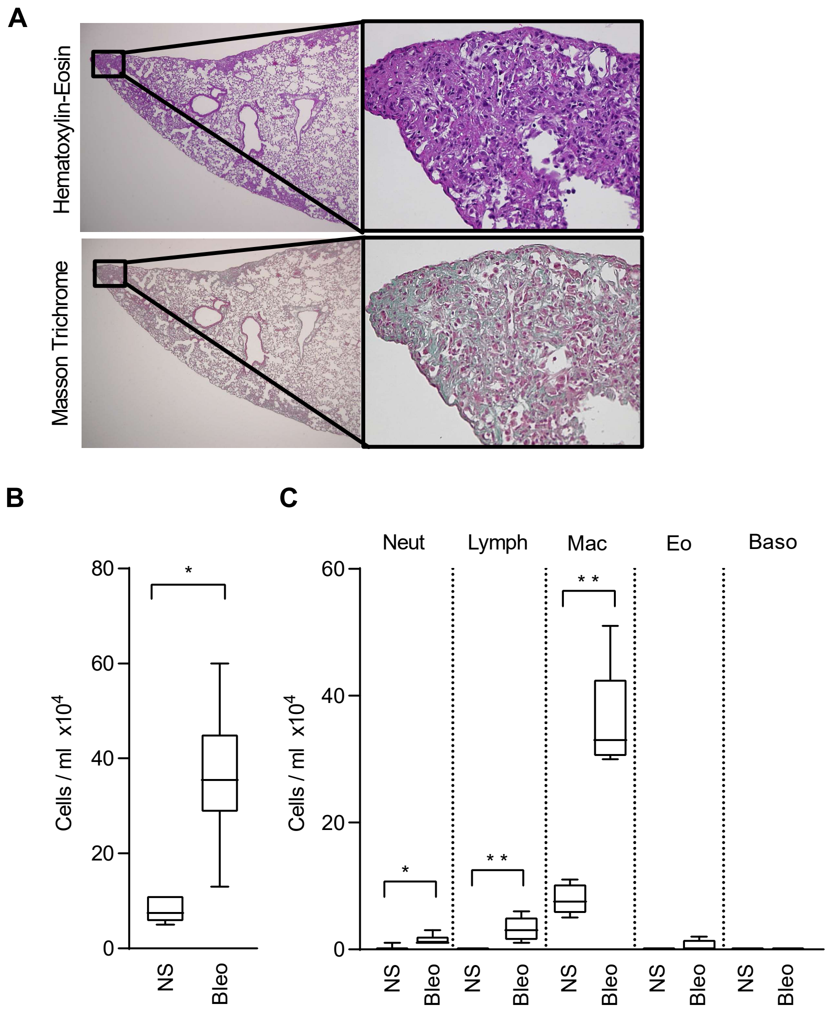

Supplement: Supplementary file 1 — Impaired Diversity of the Lung Microbione Predicts Progression of Idiopathic pulmonary Fibrosis. Table S1. Overview of DNA sequencing data. Figure S1. Comparison of the total bacterial genes between the AE and non-AE groups. Figure S2. Rarefaction curves were calculated, which suggests that all expected OTUs have been obsreved. Figure S3. the concentration of SP-A in BALF correlates well with the relative abundance of Veillonellaceae, but not serum SP-A. Figure S4. The relative abundance of family Streptococcaceae also correlated with 6MWD. Figure S5. Histopathological assesment and BAL cell counts in mice with the bleomycin-induced lung fibrosis. (PDF 7772 kb) [file 12931_2018_736_MOESM1_ESM.pdf]
